# “My Bitch Is Empty!” An Overview of the Reasons for Pregnancy Loss in Dogs

**DOI:** 10.3390/vetsci12020127

**Published:** 2025-02-05

**Authors:** George Mantziaras, Maja Zakosek Pipan

**Affiliations:** 1 Independent Researcher, Small Animal Practitioner, Kifisias Avenue 22, 15125 Marousi, Greece; gmantziaras@yahoo.com; 2 Clinic for Reproduction and Large Animals, Veterinary Faculty, University of Ljubljana, Gerbičeva 60, 1000 Ljubljana, Slovenia

**Keywords:** canine pregnancy, infertility, abortion, resorption, endometritis

## Abstract

The purpose of this review is to present reasons for pregnancy loss in dogs. Termination of pregnancy can occur at any stage of gestation, sometimes even before pregnancy is diagnosed. If embryonic death occurs before day 35 of gestation, the embryo is absorbed. Fetal death in the second half of gestation leads to abortion or fetal emphysema, maceration, or mummification. Abnormal embryonic development, competition between placental sites, or genetic abnormalities of the embryo(s) can lead to termination of pregnancy. Maternal factors that alter ovarian function or the environment in the oviducts and uterus, such as cystic endometrial hyperplasia or inflammation, can lead to pregnancy loss. Abnormalities of the placenta, uterine torsion, metrocoele, environmental stress, and malnutrition can have detrimental effects on fetal development. Several drugs or medications can also have embryotoxic/teratogenic effect. Endocrinological disorders such as hypoluteodism, gestational diabetes, and hypothyroidism can be reasons for pregnancy failure. Several bacteria, protozoa, and viruses can cause pregnancy loss in dogs. Etiological diagnoses of pregnancy loss in dogs are challenging as the exact causes are not always obvious; so, a multi-faceted approach is required.

## Simple Summary

This review examines the causes of pregnancy loss in dogs, which can occur at any stage of gestation, sometimes even before pregnancy is confirmed. Early embryonic death, especially before 35 days, often results in resorption of the embryo, while fetal death in later stages can lead to miscarriage or conditions such as emphysema, maceration, or mummification. Contributing factors include abnormal embryonic development, genetic defects, and competition between placental spaces. Maternal factors such as uterine abnormalities (e.g., cystic endometrial hyperplasia or inflammation), placental problems, uterine torsion, nutritional deficiencies, and environmental stressors can also affect the viability of the pregnancy. Hormonal imbalances, including hypoluteodism and hypothyroidism, and infections caused by bacteria, viruses, or protozoa further complicate the outcome of pregnancy. Given the complexity of these cases, accurate diagnosis often requires a comprehensive, multi-faceted approach to identify the underlying causes and improve reproductive management in dogs.

## 1. Introduction

Veterinarians specializing in reproduction often find themselves in the difficult position of telling owners and breeders that their bitch is not pregnant. The purpose of this overview is to document possible causes, factors, and pathological conditions that can lead to premature termination of pregnancy in dogs.

According to Phemister [1], the prenatal development of the dog is divided into three periods. Fertilization is followed by the ovum period (days 2–17 after fertilization), which is characterized by the free-floating blastocyst in the uterine tube that migrates to the uterus. On day 19 after implantation of the blastocyst, the embryonic period begins, which ends on day 35. From day 35, the fetal period begins, during which characteristic features appear and the fetus can be recognized as a canine. The fetal period ends at parturition [1,2].

Pregnancy loss can occur in dogs at any stage of gestation. Early death of embryos refers to loss of conceptus in the early stages of pregnancy, when embryos are free-floating in the oviducts and later in the uterus, or at implantation when attachment to the uterus occurs [3]. Canine pregnancy can be diagnosed with ultrasonography as early as 19 days after the luteinizing hormone (LH) surge [4,5,6,7,8]. If embryonic death occurs at this early stage before the 19th day, it is most likely not be diagnosed. Therefore, it is very difficult to determine the exact incidence of early embryonic loss.

Anything that can alter the normal oviductal or uterine environment (for example inflammation, infection, or hormonal changes) can lead to early embryonic death. Development of the embryos before implantation depends heavily on the environment within the oviducts and uterus [9]. In female dogs with cystic endometrial hyperplasia, early embryonic loss is more common as the uterine environment is abnormal and facilitates bacterial invasion and growth, while the thickened endometrium does not allow normal attachment of the conceptus and the development of fetomaternal communication. The administration of antibiotics post-mating to prevent early death has been shown to be of little value [10]. Other reasons for early embryonic death include genetic and chromosomal abnormalities of the embryo(s) [9,11,12].

**Figure 1:**
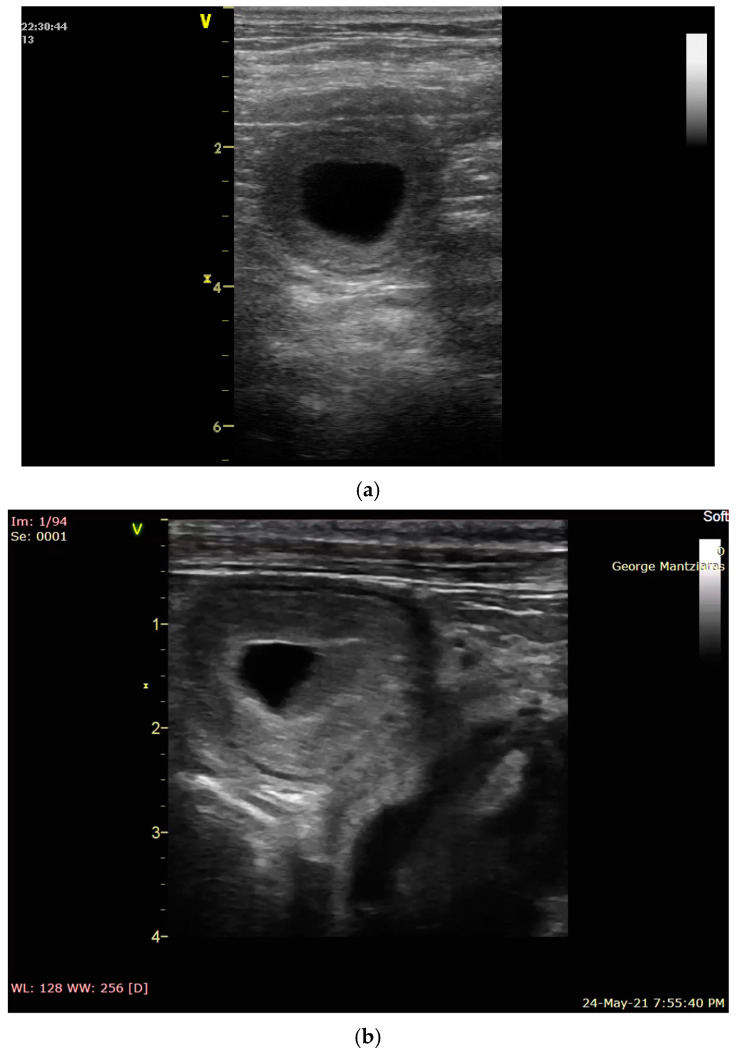
(**a**,**b**) Empty gestational sacs due to early embryonic death.

When pregnancy ends between days 19–35, the embryo(s) is (are) absorbed; this phenomenon is called resorption (Figure 1). If fetal death occurs later, in the second half of the pregnancy, an abortion occurs. Abortion is the expulsion of a dead or non-viable fetus [2,9,13].

**Figure 2:**
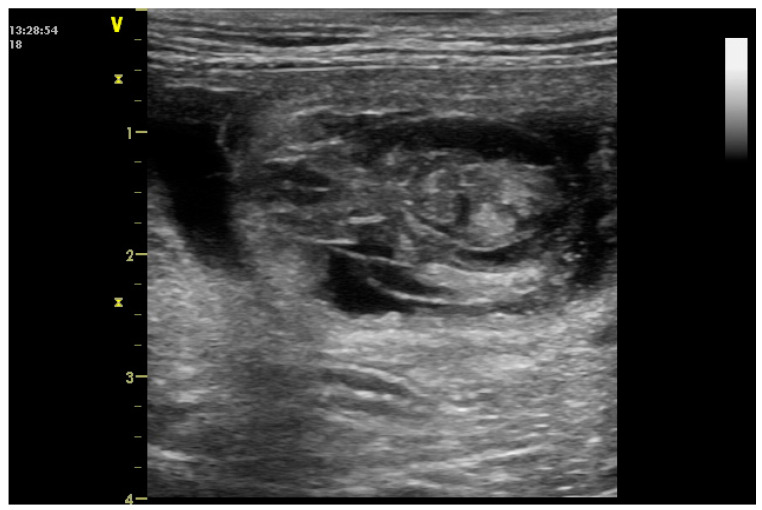
Dead embryo, around 33rd–35th day of pregnancy.

If a fetus dies during the later stages of pregnancy and is not expelled, it may develop emphysema and maceration, causing the mother to potentially exhibit signs of toxemia or sepsis. Alternatively, if the fetus is neither aborted nor macerated, it could become mummified [11] (Figure 2). Fluid is reabsorbed into the bitch’s circulation; tissues become dehydrated and autolytic and the placenta dissolves. For the mummified fetus not to be aborted, at least initially, the uterus must contain normal fetuses and a persistent corpora lutea [14].

The actual incidence of resorption rates in dogs is not easy to determine as it can only be diagnosed by ultrasound or macroscopic examination of surgically removed uterine tracts [15]. The percentage of affected conceptions (incidence of resorption expressed as number of resorption sites at different stages among total counted conceptuses) and the percentage of pregnancies affected by resorption show wide variation in the literature. Embryonic resorptions were recorded in 4.8% of pregnant bitches with no history of reproductive disease and in 5.8% of bitches that had previously failed to become pregnant [6]. According to other studies, at least one site of embryonic resorption was observed in 48.3% (42/87), 30% (6/20), 42.9% (33/77), and 32.6% (43/132) of pregnancies studied [15,16,17,18]. The incidence of resorption has been reported at 14.2% (61/431), 10.6% (14/132), and 17.3% (87/504) [15,16,17].

## 2. Reasons for Pregnancy Losses in the Bitch

The potential reasons of pregnancy failure in dogs can be categorized as follows:

1. Endocrine disorders;
2. Infectious causes:
  - Bacterial causes;
  - Protozoal causes;
  - Viral causes;
3. Uterine pathology;
4. Exogenous drugs;
5. Age;
6. Congenital defects and genetic disorders;
7. Other conditions.

### 2.1. Endocrine Disorders

#### 2.1.1. Hypoluteoidism

In the bitch, the only source of progesterone synthesis is the corpora lutea [19,20,21]. Insufficient production of progesterone by the corpora lutea (CL), a condition named hypoluteodism, has been described as a potential cause of abortion in bitches [22]. Thuróczy et al. (2016) [23] reported that serum progesterone concentrations in bitches that aborted were significantly lower from the third until the eighth week post ovulation, compared with bitches with normal pregnancy and parturition. A premature decline in progesterone serum concentration (below 2 ng/mL for more than 24 h) terminates pregnancy [24,25]. German shepherds seem to be predisposed [26]. Age and pregnancy rank are reported to be positively correlated with the risk for luteal insufficiency [27].

The cause of hypoluteoidism is still unclear. It may be a primary defect of the corpora lutea or premature luteolysis triggered by the production of prostaglandins as a result of inflammation, such as endometritis. According to Gunzel-Apel et al. (2006) [26], low relaxin production by the placenta can reduce the concentration of prolactin. Prolactin is the primary luteotropic hormone in the second half of pregnancy [28]. Thus, when placental function is impaired and the concentration of relaxin is low, insufficient prolactin production may occur [22,26]. It has been suggested, although not definitively proven, that disturbances in prolactin production may contribute to hypoluteoidism. Prolactin is important for supporting the corpus luteum, which produces the progesterone required to maintain pregnancy in the second half of gestation. In pregnant bitches, plasma prolactin levels increase approximately one month after ovulation, accompanied by a decrease in plasma progesterone levels. Even in healthy cyclic bitches, prolactin secretion is most pronounced in the second half of the luteal phase, suggesting that declining progesterone levels during this period may influence prolactin secretion [29]. In addition, fetal death, regardless of outcome, may also contribute to a decline in progesterone levels in the bitch. The presence of progesterone antibodies in pregnant bitches has also been mentioned as a possible factor in the pathogenesis of hypoluteoidism [30].

Hinderer et al. (2021) measured serum progesterone concentrations in pregnant bitches during early, mid, and late pregnancy and concluded that progesterone concentrations may have been lower than previously described as adequate in the literature. Bitches with serum progesterone concentrations lower than 6.68 ± 2.18 ng/mL showed no signs of abortion or distress, naturally giving birth to healthy puppies at the expected time [31].

Several recommendations for the diagnosis of hypoluteodism have been published in the literature [27,30,32,33,34,35]. Natural or synthetic progestins have been proposed for the management of hypoluteoidism: medroxyprogesterone acetate (0.1 mg/kg per os SID) [32], altrenogest (0.088 mg/kg per os SID) [36], progesterone in oil (1–2 mg/kg, IM, every other day) [26], and micronized progesterone (10 mg/kg po BID [9] or 100 mg/30 kg TID [27].

Synthetic progestins have a higher risk potential for side effects and their use is not recommended [9]. Early supplementation of progesterone can cause several side effects in both the dam and the puppies, such as dystocia, pyometra and resulting septicemia, masculinization of female fetuses, prolonged gestation with fetal death, congenital heart defects, facial deformities, limb reduction, and hypospadias in male fetuses, as well as cryptorchidism [19,22,31,32,33,34]. Treatment should therefore begin as late as possible. Administration of progesterone should be stopped as early as the 58th day of pregnancy, as this can otherwise lead to prolonged gestation and reduced milk production at the time of whelping. The viability of the fetuses and correct organogenesis should be checked with ultrasonography before the interruption of treatment. In 2024, Egyptien et al. (2024) reported an interesting case of glucocorticoid-like effects in a pregnant Irish Setter bitch supplemented with micro-ionized progesterone [37]. A similar effect in humans was described by Harte et al. (1997) [38].

In the authors’ practice, receptive bitches are monitored weekly after day 30 to 35 post-ovulation, and micronized progesterone (initial dose of 10 mg/kg orally BID) is administered when the serum progesterone concentration is lower than 5 ng/mL before day 58 post-ovulation. On days 58 and 59, progesterone is administered at half dose once daily to mimic the natural fall in progesterone, after which no more progesterone is administered.

The actual incidence, diagnosis, and consequences of hypoluteoidism during pregnancy remain controversial. Further clinical research is needed to avoid overdiagnosis by veterinarians.

#### 2.1.2. Gestational Diabetes

Canine gestational diabetes mellitus is a rare clinical condition with few reports in the literature [39,40,41,42], characterized by elevated blood glucose levels and glucose in the urine because of low insulin secretion and increased peripheral insulin resistance. Insulin resistance is promoted by progesterone and by the progesterone-induced secretion of growth hormone from the mammary glands, which is considered to have an anti-insulin effect [43,44,45]. Gestational diabetes typically occurs in the second half of pregnancy when the demand for insulin increases, and it often resolves within a few days after parturition.

Gestational diabetes usually affects neonatal survival and is not a cause of resorption or abortion. Common clinical signs in the bitch include polydipsia, polyuria, weight loss despite increased appetite, and lethargy.

Medical management of pregnant bitches should begin as soon as possible to increase the success rate, as follows:

- Feeding a diet low in simple carbohydrates helps maintain stable blood glucose levels. Feeding the dog with small, more frequent meals helps keep blood sugar levels steady. Specific veterinary-prescribed therapeutic diets designed for diabetic dogs can also be used;
- Most dogs with gestational diabetes require insulin administration. Insulin dosage depends on blood glucose levels; therefore, regular monitoring is essential to adjust insulin doses appropriately and avoid hypoglycemia. Frequent monitoring is crucial throughout pregnancy as hormone levels fluctuate and insulin needs may change;
- In cases where diabetes is complicated with ketoacidosis, medical management should include fluid therapy, correction of acidosis, restoration of renal function, and delivery of insulin to its tissue receptors.

Pregnancy should be carefully monitored with regular ultrasounds to evaluate fetal health, as gestational diabetes can increase the risk of complications such as larger-than-normal puppies (macrosomia), stillbirths, or premature births. Labor should also be closely supervised by the veterinarian in case of potential complications. In cases where the puppies are too large or there are concerns for the mother’s health, cesarean section should be performed. Blood glucose levels should be also monitored after parturition to ensure that the diabetes has been resolved and to avoid unnecessary insulin administration that may cause hypoglycemia, with symptoms that may include weakness, disorientation, tremors, and seizures [40,45,46,47,48]. Postpartum spaying is often recommended to prevent future occurrences.

#### 2.1.3. Hypothyroidism

Although hypothyroidism is the most diagnosed endocrinopathy in dogs, there is limited and controversial information linking this condition to fetal loss, and the role of hypothyroidism in canine pregnancy may be overestimated. Hypothyroidism is known to cause several metabolic and systemic effects, including weight gain, lethargy, and reproductive disorders. However, the direct effects on fertility and the course of pregnancy remain unclear. Some studies suggest that untreated hypothyroidism can lead to infertility, irregular estrus cycles and, in severe cases, fetal resorption or abortion [22,49,50,51,52,53,54,55]. However, other studies did not find a significant link between hypothyroidism and pregnancy complications [56,57,58,59,60], leading to debate in the veterinary community. Therefore, it is important to consider other potential causes of reproductive failure in dogs, such as infections, genetic factors, and environmental stressors, rather than attributing pregnancy loss solely to thyroid dysfunction. Further research is needed to substantiate cause and effect.

### 2.2. Infectious Causes

#### 2.2.1. Bacterial Causes

##### *Canine* *brucellosis*

*Brucella canis* (*B. canis*) infects dogs and wild canidae causing abortions, reproductive failure, and persistent infection with intermittent bacteremia. Human infections have been reported after laboratory accidents or after contact with infected dogs, and though *B. canis* has the lowest zoonotic potential among the classic Brucella spp., it has been diagnosed in several countries worldwide [61,62,63,64,65,66,67].

*B. canis* is transmitted via oral, nasal, conjunctival, or genital mucosa after contact with contaminated material, tissue, or fluid (placental material or aborted fetuses, urine, milk, male and female genital secretions). Huge numbers of bacteria are shed into the environment with aborted material. Venereal transmission is also very important, as *B. canis* is shed in vaginal discharge and in the semen of infected males, particularly during the first 2 months after infection. Dogs can shed *B. canis* intermittently for up to 2 years at lower concentrations in semen [62,63,68]. *B. canis* causes persistent, intermittent bacteremia, and it may be transmitted via blood transfusions [63]. Vertical intrauterine infection of puppies can occur. Those puppies that are born may become infected through milk during suckling, and/or by contact with aborted material [69]. Puppies that survive may play an important epidemiological role, as they become permanent carriers of *B. canis* [70].

Enlargement of the lymph nodes may be the only symptom in infected dogs. Infected bitches usually show lesions typical of Brucella spp, such as metritis, placentitis, and abortion, with focal necrosis of the chorionic villi and many bacteria in the trophoblast cells [71,72,73]. The aborted fetuses may show signs of bronchopneumonia, myocarditis, renal hemorrhage, lymphadenitis, and hepatitis [71]. Most abortions occur between the 45th and 55th day of gestation [71], and also in the early stages of gestation (between 10 and 35 days) in some cases [62,71]. The disease can manifest itself in interrupted whelping [74], repeated abortions, or abortions followed by normal litter. After an abortion, a long-lasting serious or sometimes viscous and gray-green discharge from the vagina is often observed, lasting up to 6 weeks [65,71]. Stillbirths, weak puppies, and increased neonatal death have also been reported in the literature [69,70,71].

Clinical manifestations of *B. canis* in males include loss of libido, enlargement of the submandibular and retropharyngeal lymph nodes, painful scrotal enlargement, epididymitis, orchitis, testicular atrophy in chronic cases, and prostatitis. Semen analysis shows reduced ejaculate volume and poor semen quality with many sperm defects and head-to-head agglutinations, while chronic cases may be oligospermic or azoospermic [68,69,75,76]. The most common non-reproductive manifestations of *B. canis* infection are chronic uveitis, hyperpigmentation of the iris, inflammatory infiltrates in the vitreous, multifocal chorioretinitis, and discospondylitis [77,78,79].

The standard diagnostic test to confirm *B. canis* requires a culture of vaginal discharge, semen, aborted fluids/tissues, urine, or blood, and it is known that bacteremia persists for 2–4 weeks [62,63]. Samples from vaginal and uterine secretions should be taken during proestrus–estrus or from bitches that have aborted. Semen from dogs collected 3 months after infection may have low, few, or even no concentration of bacteria and cultures may therefore be negative. A negative culture should not rule out infection. Isolation of *B. canis* has low sensitivity, with many false negative results. Antimicrobial treatment, the use of EDTA to inhibit bacterial growth, and improper conditions for the storage and transportation of samples further increase the low sensitivity and additional diagnostic methods are always recommended [72]. Diagnosis can also be determined by detecting *B. canis* DNA in whole blood, semen, or tissue samples using polymerase chain reaction (PCR) [80]. False negative results can occur in the absence of bacteremia, when antimicrobial drugs have been taken, or when PCR inhibitors (heparin) are present in blood samples. Serologic tests are useful and can be used as a first-line screening test. The most used serologic tests are the rapid slide agglutination test (RSAT) [81] and the rapid slide agglutination test with 2-mercaptoethanol 2MERSAT [82]. Cross reactions with several other bacteria can lead to false positive results [62]. The tube agglutination test (TAT) is used to confirm positive RSAT or 2ME-RSAT results. The agar gel immunodiffusion test (AGID) can also be used, but it has significant disadvantages including cross reactions and subjectivity in the interpretation of precipitin lines [67,83]. Enzyme-linked immunosorbent assay (ELISA) has also been used for the diagnosis of *B. canis*, but the sensitivity and specificity vary depending on the antigen used [83,84]. Indirect ELISA is more specific and less sensitive than TAT, but more sensitive than agglutination methods and AGID [67]. ELISA can detect antibodies in chronically infected dogs with negative 2ME-RSAT and AGID results [85] and can be used as a confirmatory test [83,86]. In all cases of negative serological results and regardless of the method used, dogs should be tested at least twice at 30-day intervals, as false negative results are common in the first 3 to 4 weeks after infection, even if bacteremia is present.

Treating infected dogs is difficult, time-consuming, and with questionable results [87]. The proposed antimicrobial protocols are a combination of doxycycline with tetracycline or enrofloxacin with streptomycin, which have a synergistic effect in vitro [88]. Administration of oxytetracycline for 4 weeks and streptomycin in the 1st week of treatment has been shown to be effective in 79% of dogs [89]. Ovariohysterectomy or orchiectomy should be recommended to minimize the spread of bacteria in secretions.

Breeding kennels with a diagnosed case should test all animals and isolate the infected ones. Dogs from infected kennels should not be sold or used for breeding or semen banking. Confirmed cases of *B. canis* should be reported to the responsible authorities, depending on the country.

As a preventative measure, all stud dogs and bitches in the kennel should be tested once or twice a year. New dogs should be isolated until they have tested seronegative twice at one-month intervals [63]. There is no vaccine available.

##### *Campylobacter* *jejuni*

*Campylobacter jejuni* is a bacterium that is frequently found in ruminants and has only been detected in a limited number of cases of canine abortions worldwide [90,91,92]. Transmission can occur from animal to animal, and from animals to humans, posing a zoonotic risk. An emerging concern is that organically suitable raw food (BARF), if not properly prepared or stored, can serve as a source of infection [93]. *C. jejuni* infections of the canine reproductive tract can occur either by ascending infection or by blood-borne transmission [93]. Clinical signs in pregnant dogs may include abortions around the 45th day of gestation or the birth of weak or stillborn puppies. In some cases, affected bitches show no outward signs of disease, while others may exhibit a profuse, odorless, hemorrhagic vaginal discharge. In addition, adult dogs may show gastrointestinal symptoms such as diarrhea and vomiting [90,91,92,94].

Diagnosis is usually confirmed by PCR or culture, with samples taken from the placenta, fetal stomach, lungs, liver, or cranial vagina of the infected bitch [95]. Treatment options include the administration of antibiotics such as macrolides and fluoroquinolones, which have been shown to be effective against the bacterium [94]. While *C. jejuni* is not a common cause of abortion in dogs, its potential zoonotic transmission, particularly through raw food, emphasizes the importance of proper food processing and hygiene practices to mitigate risk.

##### *Salmonella* spp.

Salmonellosis is not a common cause of pregnancy failure in dogs. It is transmitted via the fecal–oral route, as the bacteria are excreted in the feces and through contact with fomites [96]. Reproductive disorders usually follow an enteric or systemic disease. Serotypes of *S. enterica*, especially Typhimurium, Panama, and Montevideo, mainly affect dogs [94].

Very few cases of abortion due to samlonellosis have been described in the literature. S. panama has been cultured from aborted puppies [97] and *S. enterica* subsp. Houtenae has been isolated in two recent cases of abortion [98,99]. In most cases, infected animals show no symptoms. Clinical signs include systemic disease in the female and late-term abortion on approximately the 52nd to 56th day of gestation [97,99,100]. *Salmonella* spp. can also cause prostatitis, orchitis, and epididymitis in male dogs [94].

Diagnosis can be confirmed via culture of the organism from fetal tissue or aborted membranes. The presence of *Salmonella* in feces can be detected by real-time PCR, and ELISA tests can also be used to detect serum antibodies [94]. Ampicillin, clavulanate-potentiated amoxycillin, gentamicin, fluoroquinolones, chloramphenicol, or trimethoprim-sulfonamides can be administered effectively [101], but the veterinarian should always remember that resistance is a common problem. The consumption of raw meat diets can play an important role in the transmission of the disease and special care should be taken if breeders prefer to feed animals raw meat or BARF [12,93].

##### *Escherichia* *coli*

*Escherichia coli* (*E. coli*) is a bacterium commonly found as part of the normal flora of the canine vagina and is the most isolated bacterium from this site [102]. It is also commonly cultured from the uterus in cases of metritis and pyometra [103,104]. *E. coli* can be transmitted by ingestion of contaminated food or water or by contact with contaminated surfaces (fomites). Although *E. coli* is a common inhabitant of the vaginal and uterine flora, very few cases of canine abortions caused by this bacterium have been reported in dogs. When abortions due to *E. coli* do occur, they usually occur between the 41st and 55th day of gestation and are often accompanied by vaginal bleeding, with signs of anemia apparent in some cases. In addition to reproductive problems, *E. coli* infections in dogs can also lead to other systemic problems, such as sepsis or urinary tract infections, especially in immunocompromised animals.

The diagnosis of an *E. coli*-related abortion is confirmed by culturing the organism from fetal tissue or aborted membranes [105,106]. In the clinical setting, *E. coli* can also be detected using molecular techniques such as PCR, which allow rapid identification. Treatment is usually with antibiotics based on culture and susceptibility testing, as *E. coli* can exhibit different resistance patterns, particularly to commonly used antibiotics. The ubiquity of the bacterium, its involvement in various infectious processes, and the potential to develop antibiotic resistance make *E. coli* an important pathogen to monitor for both reproductive and non-reproductive diseases in dogs.

##### *Streptococci* 

*Streptococci* have been found in the normal flora of healthy bitches [102,107,108,109]. However, the clinical significance of their presence remains questionable [102]. According to some studies, beta-hemolytic streptococci could be associated with vaginitis in bitches [108], while others have reported that the presence of *Streptococcus* spp. (excluding beta-hemolytic streptococci) could play a protective role, as their presence during the proestrus phase was negatively correlated with uterine infections in the luteal phase [110].

Βeta-hemolytic streptococci have been isolated from females with a history of abortion, infertility, and neonatal death. Abortions occur around the 30th to 40th day of gestation (sometimes later) [111,112,113]. Group G *Streptococcus canis*, which can be transmitted from dogs to humans via skin-to-skin contact or bite wounds, can also cause reproductive tract infections and abortions in dogs. According to a retrospective study of streptococcal infections in 393 dogs, published by Lamm et al. (2010) [114], streptococci caused fetal/neonatal septicemia and were isolated in 16 cases of abortion or neonatal death; so, in such cases, streptococcal infection should be considered in the differential diagnosis. Confirmation of the diagnosis requires histopathologic examination and routine aerobic cultures from aborted material [114].

##### *Listeria* *monocytogenes*

*Listeria monocytogenes* is a bacterial pathogen that is primarily known for infections in humans and ruminants but can also affect dogs, albeit rarely. In dogs, infections with *Listeria monocytogenes* are rare, but can occur through ingestion of contaminated food, such as raw meat or dairy products, or through contact with contaminated environments, particularly in agriculture [115]. Listeriosis in dogs can lead to a variety of clinical signs ranging from gastrointestinal symptoms such as diarrhea and vomiting to more severe consequences such as septicemia, encephalitis, and abortion.

This bacterium is of particular concern in pregnant animals. Infections during pregnancy can lead to abortion, stillbirths, or the birth of weak puppies. However, published cases of *Listeria monocytogenes* causing abortions in dogs are rare compared with other species such as cattle or sheep [116]. Most dogs tend to be asymptomatic carriers, i.e., they harbor the bacteria without showing clinical signs but can still potentially shed the organism and pose a risk to humans and other animals [117]. Sturgess [118] also reported this bacterium as a cause of abortion in a case of a systemically ill bitch with brownish vaginal discharge.

Diagnosis of listeriosis in dogs is confirmed by bacterial culture or PCR testing of blood, cerebrospinal fluid, or affected tissues. Treatment usually involves the administration of antibiotics such as penicillin or ampicillin, often in combination with aminoglycosides [115]. Despite the rarity of clinical cases in dogs, *Listeria monocytogenes* remains a pathogen of concern due to its zoonotic potential and the severity of the infections it can cause, particularly in immunocompromised individuals or pregnant animals.

##### *Leptospirosis* 

*Leptospirosis* is a bacterial infection caused by various serovars of the genus Leptospira and has a significant impact on reproduction in dogs. Dogs serve as maintenance hosts for the Leptospira serovars icterohemorrhagiae, canicola, and grippotyphosa. They can also serve as intermediate hosts for serovars such as autumnalis, australis, tarasovi, bolum, bataviae, and Bratislava [119]. Infections with the Bratislava serovar have been associated with infertility and abortion in breeding kennels [120].

*Leptospirosis* is usually transmitted through contact with the urine of infected animals, which contaminates water, soil, or other surfaces. The bacteria enter the body through mucous membranes or injured skin. Transmission is primarily through urine, but vaginal transmission has also been reported, although this is rare [120]. Infections during pregnancy can lead to late abortions, stillbirths, or the birth of weak puppies. Dogs may show few to no clinical signs prior to these reproductive problems, but in some cases, signs such as fever, jaundice, or kidney and liver dysfunction may occur [121].

In male dogs, leptospirosis can lead to orchitis, which can result in infertility, further complicating breeding programs [122]. Diagnosis is confirmed by serology, PCR, or bacterial cultures from urine, blood, or fetal tissues. Antibiotic therapy, particularly with doxycycline, is used to eradicate infection and reduce the risk of carrier status and zoonotic transmission [121]. Regular vaccination is crucial to prevent infection, particularly in areas where leptospirosis is endemic. The disease is zoonotic, meaning it can be transmitted to humans, which emphasizes the importance of proper hygiene and preventative care in affected dogs [123].

##### *Mycoplasma* 

*Mycoplasma*s are recognized as part of the normal vaginal flora in dogs [102,109,124,125] and can be associated with urogenital tract infections, purulent endometritis, poor conception rates, early embryonic death, fetal resorption, abortion, stillborn pups, and neonatal deaths [124,126,127,128,129,130]. In utero transmission and infection has been described [131]. *Mycoplasma* can be transmitted by sexual intercourse, artificial insemination, or oro-genital contact. The fetus can be infected through intra-uterine ascending infection or via the placenta [132].

The diagnosis can be confirmed by isolating the organisms from fetal tissue or uterine discharge. It should be noted that the cultivation of mycoplasma is complicated and labor-intensive [133]. Therefore, culture is not performed in daily clinical practice [102], and further investigation into its potential role in small animal reproductive pathology is required.

#### 2.2.2. Protozoal Causes

##### *Toxoplasma* *gondii*

*Toxoplasma gondii* is transmitted by congenital infection, ingestion of infected or oocyst-contaminated food or water, or the feces of an infected cat [134]. Fever, lymphadenopathy, abortus, and fetal death are the main clinical manifestations. When tachyzoites were experimentally administered to pregnant bitches, *T. gondii* caused systemic disease in the bitches and infected puppies, which died soon after birth [135].

Diagnosis is confirmed when *T. gondii* cysts are detected histologically in fetal tissue. Prevention of toxoplasmosis exposure is preferable to treatment of the disease. Feeding raw meat to dogs and contact with cat litter should be avoided [2].

##### *Neospora* *caninum*

*Neospora caninum* is a protozoan parasite that primarily infects cattle but can also have a significant impact on reproduction in dogs, which serve as the definitive hosts. *N. caninum* is known for its ability to cause transplacental transmission, which has been documented in several studies [136,137,138]. Transplacental transmission occurs when the parasite crosses the placenta during gestation and infects the developing fetus. This route of transmission is one of the main routes of infection in dogs, particularly in cases where bitches have been infected before or during pregnancy.

Reproductive complications associated with *N. caninum* infection in dogs include abortions, stillbirths, and the birth of weak or neurologically impaired puppies. Puppies infected in utero can show symptoms such as hind limb paralysis, muscle wasting, and general weakness, which may not become apparent until after birth. In some cases, infected bitches may abort repeatedly due to *N. caninum*, which is a cause for concern in breeding programs [139]. Barber et al. (1998) concluded that the frequency of vertical transmission of naturally acquired neosporosis in dogs is variable and the parasite alone does not always cause disease without concurrent postnatal infection [138].

In bitches that are carriers of the infection, there may be no obvious clinical signs during pregnancy. However, the parasite can remain inactive and reactivate during periods of stress or immunosuppression, leading to infection of the fetus and an unfavorable outcome for the pregnancy [140]. Diagnosis of *N. caninum* is usually confirmed by serologic testing, PCR, or histopathologic examination of aborted tissue, although detection of the organism in placental tissue is critical for confirmation of transplacental transmission.

Unfortunately, there is no definitive treatment that can prevent transplacental transmission of *N. caninum*. Treatment strategies essentially focus on preventing exposure to the parasite, e.g., by controlling access to raw meat and ensuring that dogs do not feed on the remains of infected animals [141]. Research into vaccine development is ongoing, but no commercial vaccines for dogs are currently available.

##### Leishmaniasis (*Leishmania infantum*)

Leishmaniasis caused by *Leishmania infantum* is primarily a vector-borne disease transmitted by the bites of infected sandflies. Although the primary manifestations in dogs include cutaneous and visceral forms, reproductive complications have increasingly been recognized. A notable case of placentitis associated with leishmaniasis in dogs was reported by Dubey in 2005 [142], in which the primary lesion was necrosis and mixed leukocyte infiltration was observed within the placenta. Numerous Leishmania amastigotes were detected in the placental trophoblasts, confirming parasitic involvement in the reproductive pathology.

Although placentitis due to *Leishmania infantum* is rare, transplacental transmission of the parasite has been documented, which can lead to infection of the fetus. This vertical transmission can lead to abortion, stillbirths, or the birth of infected puppies that are likely to develop clinical leishmaniasis later in life. Puppies born to infected mothers may show signs of visceral leishmaniasis, such as lymphadenopathy, splenomegaly, cachexia, and skin lesions, although some may remain asymptomatic carriers for a prolonged period [143].

Leishmaniasis poses a major challenge in breeding programs. Infected bitches can carry and transmit the parasite transplacentally or venereally, making disease control difficult, especially in endemic areas. The exact prevalence of reproductive manifestations such as abortion or placentitis due to leishmaniasis in dogs is not well understood, but the ability of the parasite to infect a variety of cells, including macrophages and trophoblasts of the placenta, is thought to contribute to these rare but serious reproductive problems [144].

The diagnosis of leishmaniasis in reproductive cases is usually made via histopathologic examination of the placenta and fetal tissue, which can identify Leishmania amastigotes. PCR tests and serologic testing can also be used to detect the parasite in both the mother and the fetus [145]. Treatment of infected bitches is complex and often involves the use of antileishmanial drugs such as allopurinol or miltefosine, although these drugs may not completely eliminate the parasite and thus pose a risk of continued transmission [143].

#### 2.2.3. Viral Causes

##### Canine Parvovirus Type 1

Canine parvovirus type 1 (CPV-1), also known as canine minute virus (CnMV), was first detected in the feces of asymptomatic dogs in 1967 [146]. Unlike the more familiar canine parvovirus type 2 (CPV-2), CPV-1 usually causes subclinical or mild infections in adult dogs but can have serious effects on reproductive health and newborns. While many dogs infected with CPV-1 show no signs of disease, others may develop respiratory distress, intestinal inflammation, or even neurological symptoms [147,148]. The virus is associated with an increased mortality rate in neonates, particularly in puppies younger than four weeks of age, where it can cause a range of symptoms from hemorrhagic enteritis, depression, and anorexia to more severe symptoms such as acute myocarditis and respiratory distress [149,150,151].

Reproductive effects of CPV-1 have been documented, and the virus can cause transplacental infections. Depending on the stage of pregnancy during infection, the consequences can range from embryonic resorption and abortion to birth defects and neonatal death [152]. Carmichael et al. (1991) [153] reported that CPV-1 led to fetal infections resulting in abortions or malformations. Delayed fetal development has also been observed in cases where fetuses were infected late in gestation [154]. However, the overall incidence of reproductive failure due to CPV-1 is relatively low and only one confirmed case of abortion after natural infection has been reported in the literature [155].

Diagnosis of CPV-1 can be difficult due to its often mild or asymptomatic course, but PCR and immunofluorescence tests can detect the virus in fetal or neonatal tissues such as heart muscle, intestine, and lung [156,157]. These diagnostic tools are essential to confirm cases of CPV-1-related reproductive loss.

A vaccine against CPV-1 is available and vaccination of breeding animals is recommended to prevent reproductive complications associated with the virus [158]. Although CPV-1 is less pathogenic than CPV-2, it can cause significant morbidity and mortality in newborns, as well as reproductive losses, making vaccination and early detection critical in breeding programs.

##### Canine Herpesvirus 1α

Canine herpesvirus 1 (CaHV-1) has been shown to be widespread in domestic dog populations. The virus is transmitted through direct contact with genital or oronasal secretions of infected animals. Newborns can also be infected during their passage through the birth canal [154].

CaHV-1 infection can be the reason for the loss of young puppies. It was first isolated from stillborn puppies by Carmichael in 1964 [159]. The fatal form of the disease usually occurs in puppies younger than 2 weeks of age [160], with symptoms of generalized disease such as loss of appetite, abdominal pain, diarrhea, ataxia, serosanguineous nasal discharge, and mucosal bleeding. Death usually occurs 3 to 7 days after the onset of clinical signs and can affect an entire litter. In older puppies, it is usually a subclinical disease, while neurological disorders have also been associated with CaHV-1 infection [161].

In adult dogs, the infection is asymptomatic or with few mild symptoms from the respiratory or genital tract (typical bullous vesicles observed either in the vestibule or vagina and on the prepuce). Infected adult dogs remain healthy carriers that can be reactivated and shed the virus again [9,160].

**Figure 3:**
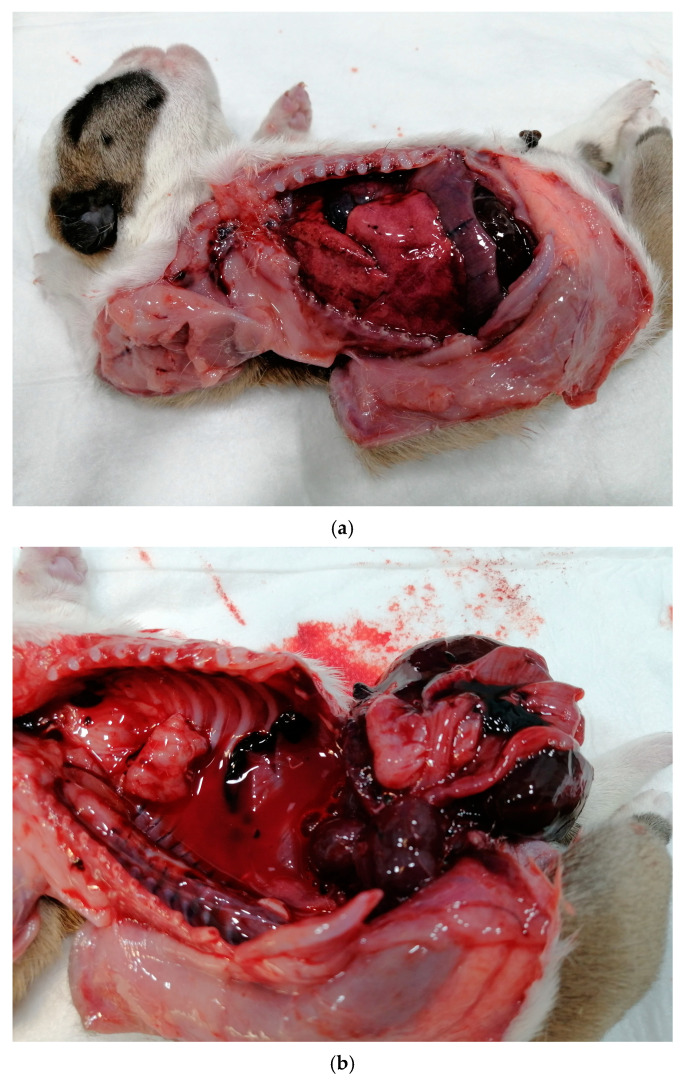
(**a**,**b**) Fluid in the thorax of a 2-day bulldog puppy with typical petechias for herpes infection. Puppy was herpes positive. (Courtesy of Kalliope Roumelioti).

The role of CaHV-1 in the outcome of pregnancy is still controversial [9,162]. It appears that transplacental infection leading to fetal or neonatal death is not common [154]. Bitches infected in mid-pregnancy develop multifocal necrotizing lesions in the placenta and may abort or deliver stillborn puppies without other clinical signs of disease or vaginal discharge. The uterus may contain dead fetuses of varying sizes. At necropsy of dead fetuses, focal necrotic lesions in the fetal organs and free fluid in the thorax are often noted, like those observed in the systemic neonatal form. Gross findings in aborted fetuses and dead neonates that are considered pathognomonic include scattered hemorrhages in the kidney, multifocal areas of necrosis in the liver and lungs, and enlargement of the spleen and lymph nodes (Figure 3) [154,163]. In conclusion, the role of CaHV-1 infection in canine pregnancy seems to have been overestimated [12,154]. There is no experimental evidence that CaHV-1 has a negative effect during early pregnancy, and it has only rarely been isolated from aborted material and from preputial and vaginal lesions in the field [164].

The diagnosis of CaHV-1 can be made from gross lesions of infected puppies and confirmed by histopathology. Diagnosis can also be made through isolating the virus from susceptible canine cell lines, via immunofluorescence testing of tissue sections or smears, or by polymerase chain reaction (PCR). Real-time PCR has also been developed for this purpose. Samples should be taken from kidneys or other affected organs, and vaginal or preputial swabs from adult dogs with genital tract lesions [12,154,165,166].

No treatment is required for adults. Canine herpesvirus-1 (CaHV-1) replicates more efficiently at temperatures below 38 °C. Keeping puppies warm helps maintain their body temperature above this threshold, which can prevent the disease from evolving. This practice reduces the virus’s ability to replicate, enhances the puppies’ immune system, and minimizes cold stress. The vaccination protocol consists of a vaccination on the 7th–10th day of gestation and a second vaccination about 5 days before parturition [167]. Another protocol recommends an additional dose 1 month before the onset of estrus [9].

##### Canine Distemper Virus

Canine distemper virus (CDV) is a highly contagious virus that affects a wide range of animals, including dogs. CDV primarily causes respiratory, gastrointestinal, and neurological symptoms, but can also have a significant impact on pregnancy and reproductive health.

Regarding its reproductive effects, CDV has been reported to cross the placenta in rare cases, resulting in infection of the fetus or stillbirth. Abortion in infected bitches can occur not only due to the direct effects of the virus on the fetus but also due to the weakened health of the mother as a result of the infection [168,169]. The effects on pregnancy can vary depending on the time of infection, with earlier infection leading to more severe consequences such as spontaneous abortion or death of the newborn [170]. Although rare, these reproductive problems highlight the importance of CDV management in breeding populations.

Diagnosis of CDV in cases of reproductive failure can be made using various methods. Post-mortem histopathology is very useful for the detection of viral inclusions and tissue damage. ELISA and PCR tests are also effective in confirming CDV infection by detecting viral antigens or nucleic acids in tissues [171]. These diagnostic tools are crucial for detecting CDV-related reproductive problems and ensuring appropriate action.

Vaccination is the most important method of preventing CDV infection and associated reproductive complications. There is an effective vaccine against CDV, and it is strongly recommended that all breeding animals are vaccinated to protect them from the virus and reduce the risk of transmission to offspring [171,172].

Routine vaccination helps maintain herd immunity and prevents outbreaks that can affect both adult dogs and their puppies.

##### Canine Adenovirus

Canine adenovirus-1 (CAV-1), also known as infectious hepatitis virus, can have a significant impact on reproduction, although it is primarily known to cause hepatitis and other systemic infections in dogs.

CAV-1 can affect pregnancy by causing abortion or the birth of weak or deformed puppies. Abortion can occur due to stress and the systemic effects of the disease on the dam [173,174]. The virus can cause a number of complications in the reproductive process, including fetal infections leading to late abortion or the birth of impaired pups. The impact on reproductive health is often related to the severity of the maternal infection and the stress it imposes on the pregnant bitch.

Diagnosis of CAV-1 infection in reproductive cases can be effectively performed using fluorescent antibody testing and PCR. These methods can detect the presence of viral antigens or nucleic acids in tissues, which is crucial for confirming the presence of the virus in cases of abortion or birth defects [175]. These diagnostic methods help to differentiate CAV-1 from other causes of reproductive failure in order to develop appropriate treatment and management strategies.

There is a vaccine against CAV-1, which is an important preventive measure. The vaccine protects against infectious hepatitis in dogs and the associated reproductive complications. It is strongly recommended that all breeding animals are vaccinated with the CAV-1 vaccine to reduce the risk of infection and subsequent reproductive problems [172]. Routine vaccination helps to maintain the overall health of the herd and prevent outbreaks of the virus.

### 2.3. Uterine Pathology

**Figure 4:**
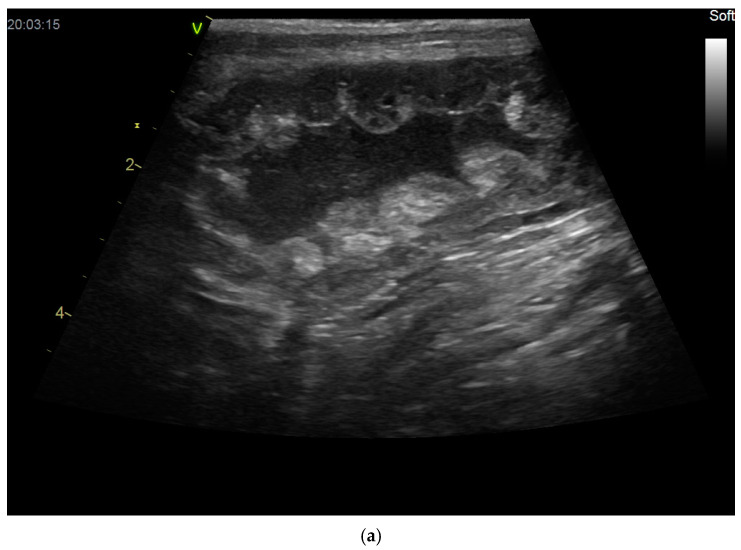

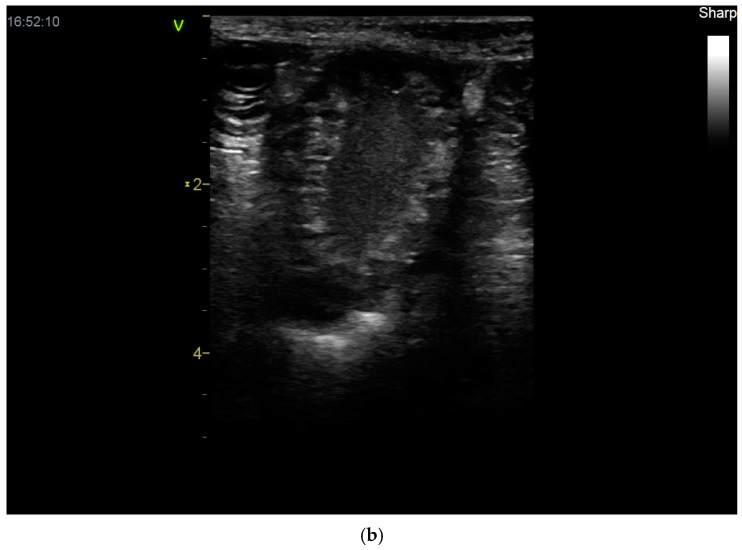
(**a**,**b**) Cystic endometrial hyperplasia with echogenic fluid in the uterine horns.

Uterine disease can lead to pregnancy loss if the normal uterine environment is altered. The most diagnosed pathological condition of the canine uterus is cystic endometrial hyperplasia (CEH), which is characterized by hyperplasia of endometrial glands that have become enlarged and cystic in appearance (Figure 4), making proper placentation and embryonic development impossible. CEH may be a possible cause of early pregnancy arrest in the bitch [9,12], and it has been found not only in older bitches [176,177,178,179] but also in young ones [180]. CEH can coexist with or predispose a bitch to pyometra, but these two pathologies may also be diagnosed independently [178]. Interestingly, despite numerous publications, the exact etiopathogenesis of this disease is still unclear [178,179,181].

The pathologic changes observed in CEH and associated syndromes, such as CEH–pyometra complex, may also affect lymphatic function within the uterine tissue. CEH is characterized by significant vascular changes including angiogenesis and increased blood flow as well as chronic inflammation, leading to edema and vascular congestion. These changes can disrupt the normal balance of tissue fluid dynamics and potentially impair the ability of the lymphatic system to drain interstitial fluid effectively. In addition, the endometrial remodeling seen in CEH is associated with fibrosis and structural changes, and this can further impair lymphatic vessel function by causing physical obstruction or altering vascular integrity [182]. While the role of the lymphatic system in CEH has not been extensively studied, the interplay between vascular and lymphatic changes associated with inflammation suggests that poor lymphatic drainage may contribute to disease progression or associated complications. Further research is needed to investigate this possible link and its clinical implications.

Another form of endometrial hyperplasia in bitches has been described, known as pseudoplacental endometrial hyperplasia (PEH). In this form, the endometrium proliferates in a highly organized manner and remodels so that it closely resembles the histology at the placentation sites during normal pregnancy. Abnormal tissue growth can disrupt the normal implantation of embryos, potentially resulting in infertility or early pregnancy loss. The hyperplastic tissue may create an environment that encourages bacterial infections such as pyometra. If pregnancy does occur, it can lead to complications like dystocia or the retention of fetal membranes [183].

Subclinical endometritis can also be an important cause of pregnancy arrest in dogs [12,184]. Mir et al. (2013) performed histopathological examinations on uterine samples from 14 bitches with unexplained infertility and from 7 bitches with unexplained pregnancy loss [166]. Uterine alterations were detected in 17 of 21 cases. In bitches with unexplained infertility, 11 of 14 cases had uterine lesions, including fibrosis with degeneration of the endometrial glands (6/11), endometritis (4/11), cystic endometrial hyperplasia (2/11), and pseudoplacental endometrial hyperplasia (PEH, 2/11). Other lesions detected were adenomyosis (1/11), mucometra (1/11), and an endometrial polyp (1/11). In bitches with previous pregnancy loss, histopathologic endometrial changes were found in six out of seven cases: endometritis (3/7), PEH (2/6), marked lymphoplasmocytic endometritis with pyometra (1/6), and CEH together with mucometra (1/6).

Gifford et al. (2014) investigated the prevalence of various lesion types detected by histologic evaluation of uterine biopsy samples from a population of 399 subfertile bitches [184]. Endometritis (170/399), cystic endometrial changes (133/399), and fibrosis (101/399) were the most common findings. Chronic endometritis with lymphocytic or lymphoplasmacytic inflammatory infiltration was observed in 89/170 cases of endometritis, while 51/170 cases showed mixed inflammatory reactions, and 30/170 were characterized as acute inflammation with neutrophils, eosinophils, or both. Uterine tissues frequently had combinations of more than one lesions present (mean of 2.7 morphological diagnoses/case). Both of these studies highlight the importance of uterine pathology in subfertile bitches.

**Figure 5:**
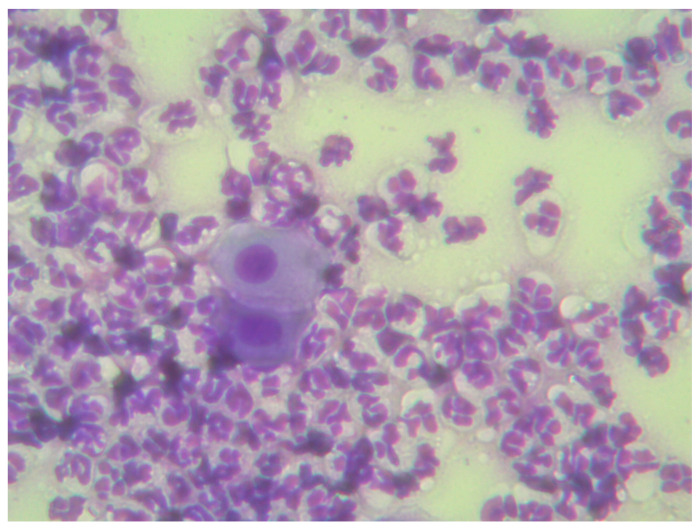
Vaginal smear from the above bitch.

Routine clinical examination, ultrasound, or blood tests are not always able to diagnose uterine pathology, especially in the early stages of the disease or when there is no fluid accumulation or uterine enlargement [178,185]. Vaginal cytology (Figure 5) in bitches with uterine inflammation may also be normal [9,166] and cannot rule out the disease on its own. The most accurate diagnostic test that can provide detailed information about the condition of the uterus is biopsy [166,184]. A uterine sample can also be taken transcervically, as reported by Christiansen et al. (2012) [186]. In dogs, unlike other species, transcervical uterine biopsy is not commonly practiced because it is relatively difficult, requires special training, and may increase the risk of uterine damage or uterine infection [166,183,186,187,188]. Another disadvantage of transcervical uterine biopsy is that diseased areas of the uterus may remain undetected [179]. Woźna-Wysocka et al. (2021) performed histological analyzes on the uteri of 120 bitches removed during routine ovariohysterectomies and concluded that in most cases, early reproductive disorders in these bitches were not confirmed by clinical signs in the examined animals. Their study suggests that despite the difficulties and risks, endometrial biopsy is a very useful tool that should be used in the investigation of uterine pathology [181]. Regarding the diagnosis of endometritis, Fontaine et al. (2009) suggested the collection of uterine samples with endoscopic transcervical catheterization as part of the investigation in all cases of unexplained infertility in bitches. A 6F catheter can be inserted into the uterus through a rigid endoscope. If needed, CO_2_ insufflation can be used to dilate the vaginal cavity and improve visualization of the cervix. The lumen can be flushed with a sterile saline solution (NaCl 0.9%, 2 mL/10 kg) and then reaspirated for further analysis. Although sedation is not required, it can facilitate the procedure. Performing this procedure during early proestrus is recommended, as the effect of progesterone on the endometrium is absent during this phase [189].

### 2.4. Exogenous Drugs

The administration of drugs required to treat maternal health issues, or the inadvertent ingestion of drugs, toxins, or hormones can be harmful to both the pregnant bitch and the embryos/fetuses. The potential effects of exogenous drugs on canine pregnancy are vast and quite complex. The administration of drugs can cause structural malformations (cleft palate, limb deformities, organ abnormalities), functional defects (issues with the nervous system, immune system, and other critical functions), growth retardation, or even pregnancy termination.

Several factors influence teratogenicity. Timing of exposure is crucial because the risk of teratogenic effects is often highest during the first period of pregnancy, when the organs are forming. The amount and length of exposure to the drug play a critical role in determining its teratogenic potential. Individual genetic factors can also influence how a drug affects the developing fetus. Examples of antimicrobials that should be avoided include aminoglycosides that can cause nephrotoxicity and ototoxicity in the fetus, fluoroquinolones that may affect developing cartilage, and tetracyclines, which can affect bone and tooth development. Antifungals such as griseofulvin and ketoconazole and chemotherapeutic agents are also associated with teratogenic effects and are harmful to developing fetuses. Glucocorticoids may induce cleft palate or other defects in puppies, as well as pregnancy loss.

There are scarce data in the literature on the safety of administering medication during canine pregnancy. Papich published a very interesting, albeit somewhat outdated, list of recommendations for a wide variety of drugs for both dogs and cats during pregnancy [190]. The use of drugs and exposure during pregnancy and lactation was summarized by Wiebe and Howard in 2009 [191]. In any case, the administration of drugs during pregnancy should be carried out very carefully, as in addition to the potentially embryotoxic/teratogenic effects, the normal physiological changes during pregnancy can also influence the availability of drugs and their distribution in the target tissues [14].

### 2.5. Age

The age of the bitch and/or the stud dog appears to affect the likelihood of early embryonic death. Gametes that are too young or too old are associated with reduced fertility in both males and females [9,192]. A recent study published by Lascialfari et al. (2023) supports this assumption, as the mean age of animals with embryo resorption at pregnancy diagnosis was significantly higher than that of animals without resorption [15].

Several studies have demonstrated the significant influence of a bitch’s age and breed on reproductive outcomes including conception rates and litter size [193,194]. Age-related changes in fertility are well documented, with bitches older than six years showing a significant decline in conception rates and litter size compared with their younger counterparts. This decline can be attributed to physiological changes such as reduced ovarian function and uterine receptivity that affect the success of conception and pregnancy. Baalbergen et al. (2021) observed that nulliparous bitches had a significantly lower conception rate compared with multiparous bitches, further emphasizing the combined influence of age and parity on reproductive success. In that study, younger nulliparous bitches often required additional measures to achieve successful conception, while older multiparous bitches had problems with increasing age [195].

In addition, breed differences have been identified as critical factors in reproductive performance. Larger breeds tend to produce larger litters due to their greater uterine capacity, while smaller breeds tend to have fewer puppies per litter. Uchańska et al. (2022) reported that maternal age and breed are the main factors in litter size, with very young (under one year) and older bitches (over six years) often producing smaller litters [196].

These findings highlight the complex interplay of age, breed and reproductive management strategies in optimizing pregnancy outcomes. Effective breeding practices should take these factors into account and apply age- and breed-specific measures, such as timing of ovulation and close monitoring during pregnancy. Further research into the mechanisms underlying these variations could increase our understanding and improve reproductive success in different dog populations.

### 2.6. Congenital Defects and Genetic Disorders

Early fetal loss, resorption, or abortion can also be the result of genetic disorders associated with abnormal phenotypic expression or congenital defects incompatible with survival. Congenital defects can be sporadic; they may occur without a known cause or as the result of intoxication. If such congenital defects occur in more than one puppy per litter or if there are repeated resorptions or abortions in a breeding pair, a different female or male should be used in the future and breeders should seek genetic counseling [9,11,14]. Genetic abnormalities have been reported to be responsible for approximately 15% of infertility cases or pregnancy loss in dog abortion [197], but it appears that the true incidence probably remains underdiagnosed [198].

### 2.7. Other Conditions

Several other causes have been reported to be responsible for pregnancy arrest in the bitch. Uterine torsion caused by abdominal trauma or during parturition can lead to embryonic death, as can metrocoele—a rare condition of inguinal, perineal, or abdominal protrusion of the uterine loop or uterine body pregnancy. Severe maternal trauma during pregnancy increases the incidence of premature placental separation and premature birth in dogs [14]. Maternal environmental stress can lead to an unfavorable uterine environment that is incompatible with fetal development [9]. Malnutrition of the pregnant bitch can also have a detrimental effect on fetal development. As in humane medicine, immune factors can also be the reason for unexplained abortions [14].

## 3. Conclusions

Diagnosing the cause of pregnancy loss in dogs is a complex and difficult task. In everyday clinical practice, the prevalence and impact of various diseases or pathological conditions can often be misjudged. Some conditions may be overestimated, leading to unnecessary worry, while others may be underestimated, leading to missed opportunities for timely intervention. As a result, the role of various factors influencing pregnancy outcome in dogs remains uncertain and requires further investigation.

Recent advances in medical research, genetics, and diagnostic technologies offer promising opportunities to improve our understanding of pregnancy disorders in dogs. Techniques such as ultrasound, endoscopy, and transcervical catheterization of the uterus have proven to be valuable tools in clinical practice. These modern diagnostic methods can provide detailed insights into the health status of the pregnant bitch and the developing fetuses.

Ultrasound-guided sampling of amniotic fluid holds considerable potential for improving prenatal diagnostics. By analyzing the biochemical parameters of fetal fluids, veterinarians can gain important information about the health status of fetuses. This approach can help to detect fetal deaths more accurately and detect hereditary diseases at an early stage [199,200]. Such advances can lead to more accurate diagnoses and better treatment strategies for pregnant dogs.

Furthermore, the integration of these advanced diagnostic techniques into routine veterinary practice emphasizes the importance of continuous education and training of veterinarians and theriogenologists. Ensuring that practitioners are adept in the proper and safe use of these technologies is critical to optimizing their utility and improving clinical outcomes.

In summary, while the diagnosis of canine pregnancy loss remains a challenge in veterinary medicine, ongoing research and technological advances provide valuable tools and insights. The future of canine prenatal care will benefit from the continued development of diagnostic techniques and the commitment of veterinarians to improving their skills and knowledge.

## Data Availability

No new data were created in this review article. All the information in provided in the reference list.

## References

[1] Phemister R.D. (1974). Nonneurogenic reproductive failure in the bitch. Vet. Clin. N. Am..

[2] Pretzer S.D. (2008). Canine embryonic and fetal development: A review. Theriogenology.

[3] Johnston S.D., Raksil S. (1987). Fetal loss in the dog and cat. Vet. Clin. N. Am. Small Anim. Pract..

[4] Yeager A., Mohammed H., Meyers-Wallen V., Vannerson L., Concannon P. (1992). Ultrasonographic appearance of the uterus, placenta, fetus, and fetal membranes throughout accurately timed pregnancy in beagles. Am. J. Vet. Res..

[5] England G.C.W., Allen W.E. (1990). Studies on canine pregnancy using B-mode ultrasound: Diagnosis of early pregnancy and the number of conceptuses. J. Small Anim. Pract..

[6] England G.C.W. (1992). Ultrasound evaluation of pregnancy and spontaneous embryonic resorption in the bitch. J. Small Anim. Pract..

[7] Beccaglia M., Alonge S., Trovo C., Luvoni G.C. (2016). Determination of gestational time and prediction of parturition in dogs and cats: An update. Reprod. Domest. Anim..

[8] Lopate C. (2023). Ultrasonography for the evaluation of pregnancy in the female canine. Reprod. Domest. Anim..

[9] Verstegen J., Dhaliwal G., Verstegen-Onclin K. (2008). Canine and feline pregnancy loss due to viral and non-infectious causes: A review. Theriogenology.

[10] England G.C., Moxon R., Freeman S.L. (2012). Delayed uterine fluid clearance and reduced uterine perfusion in bitches with endometrial hyperplasia and clinical management with postmating antibiotic. Theriogenology.

[11] Lamm C.G., Njaa B.L. (2012). Clinical approach to abortion, stillbirth, and neonatal death in dogs and cats. Vet. Clin. N. Am. Small Anim. Pract..

[12] Fontbonne A. (2023). Causes of pregnancy arrest in the canine species. Reprod. Domest. Anim..

[13] England G.C.W. (1998). Ultrasonographic assessment of abnormal pregnancy. Vet. Clin. N. Am..

[14] Johnston S., Root-Kustritz M., Olson P., Johnston S., Root-Kustritz M., Olson P. (2001). Canine pregnancy. Canine and Feline Theriogenology.

[15] Lascialfari P., Tesi M., Manetti C., Fanelli D., Rota A. (2023). Embryonic resorption rates at canine pregnancy diagnoses: A retrospective evaluation. Theriogenology.

[16] England G.C.W., Russo M. (2006). Ultrasonographic characteristics of early pregnancy failure in bitches. Theriogenology.

[17] Ortega-Pacheco A., Rodriguez-Buenfil J.C., Segura-Correa J.C., Montes de Oca Gonzalez A.R., Jimenez-Coello M. (2006). Prevalence of fetal resorption in stray dogs in Yucatan, Mexico. J. Small Anim. Pract..

[18] Totton S.C., Wandeler A.I., Gartley C.J., Kachhawaha S., Suman M., Ribble C.S., Rosatte R.C., McEwen S.A. (2010). Assessing reproductive patterns and disorders in free-ranging dogs in Jodhpur, India to optimize a population control program. Theriogenology.

[19] Tsutsui T. (1983). Effects of ovariectomy and progesterone treatment on the maintenance of pregnancy in bitches. Jpn. J. Vet. Sci..

[20] Okkens A.C., Dieleman S.J., Bevers M.M., Willemse A.H. (1985). Evidence for the non-involvement of the uterus in the lifespan of the corpus luteum in the cyclic dog. Vet. Q..

[21] Verstegen-Onclin K., Verstegen J. (2008). Endocrinology of pregnancy in the dog: A review. Theriogenology.

[22] Johnson C.A. (2008). High-risk pregnancy and hypoluteoidism in the bitch. Theriogenology.

[23] Thuróczy J., Müller L., Kollár E., Balogh L. (2016). Thyroxin and Progesterone Concentrations in Pregnant, Nonpregnant Bitches, and Bitches during Abortion. Theriogenology.

[24] Concannon P.W., Hansel W. (1977). Prostaglandin F2 Induced Luteolysis, Hypothermia, and Abortions in Beagle Bitches. Prostaglandins.

[25] Kowalewski M.P. (2012). Endocrine and Molecular Control of Luteal and Placental Function in Dogs: A Review. Reprod. Domest. Anim..

[26] Gunzel-Apel A.R., Zabel S., Bunck C.F., Dieleman S.J., Einspanier A., Hoppen H.O. (2006). Concentrations of progesterone, prolactin and relaxin in the luteal phase and pregnancy in normal and short-cycling German Shepherd dogs. Theriogenology.

[27] Rosset E., Mazereaux C., Buff S. Retrospective study of hypoluteoidism cases in the bitch. Proceedings of the 2010 EVSSAR Congress.

[28] Concannon P., Tsutsui T., Schille V. (2001). Embryo development, hormonal requirements and maternal responses during canine pregnancy. J. Reprod. Fertil..

[29] Gutierrez Rufo J., Gazzano A., Mariti C. (2017). Prolactin in Female Domestic Dogs: A Mini-Review. Mathews J. Vet. Sci..

[30] Krachudel J., Bondzio A., Einspanier R., Einspanier A., Gottschalk J., Kuechenmeister U., Muennich A. (2013). Luteal Insufficiency in Bitches as a Consequence of an Autoimmune Response against Progesterone?. Theriogenology.

[31] Hinderer J., Lüdeke J., Riege L., Haimerl P., Bartel A., Kohn B., Weber C., Müller E., Arlt S.P. (2021). Progesterone Concentrations during Canine Pregnancy. Animals.

[32] Görlinger S., Galac S., Kooistra H.S., Okkens A.C. (2005). Hypoluteoidism in a bitch. Theriogenology.

[33] Tibold A., Thuróczy J. (2009). Progesterone, Oestradiol, FSH and LH Concentrations in Serum of Progesterone-Treated Pregnant Bitches with Suspected Luteal Insufficiency. Reprod. Domest. Anim..

[34] Kustritz M.V., Concannon P.W., England G., Verstegen J., Linde Forsberg C. (2001). Use of Supplemental Progesterone in Management of Canine Pregnancy. Recent Advances in Small Animal Reproduction.

[35] Kustritz Root M.V. (2005). Pregnancy Diagnosis and Abnormalities of Pregnancy in the Dog. Theriogenology.

[36] Estill C.T. (1998). Vet Med Today: Theriogenology Question of the Month. J. Am. Vet. Med. Assoc..

[37] Egyptien S., Somville M., Deleuze S. Progesterone supplementation during canine pregnancy: Friend or foe?. Proceedings of the 2024 EVSSAR Congress.

[38] Harte C., Henry M.T., Murphy K.D., Mitchell T.H. (1997). Progestogens and Cushing’s syndrome. Ir. J. Med. Sci..

[39] Norman E.J., Wolsky K.J., MacKay G.A. (2006). Pregnancy-Related Diabetes Mellitus in Two Dogs. N. Z. Vet. J..

[40] Fall T., Johansson Kreuger S., Juberget A., Bergström A., Hedhammar Å. (2008). Gestational Diabetes Mellitus in 13 Dogs. J. Vet. Intern. Med..

[41] Armenise A., Pastorelli G., Palmisano A., Sontas H.B., Romagnoli S. (2011). Gestational Diabetes Mellitus with Diabetic Ketoacidosis in a Yorkshire Terrier Bitch. J. Am. Anim. Hosp. Assoc..

[42] Yoon W., Suh S., Hyun C. (2015). Gestational Diabetes in a Yorkshire Terrier Dog. J. Vet. Clin..

[43] Eigenmann J.E., Eigenmann R.Y., Rijnberk A., van der Gaag I., Zapf J., Froesch E.R. (1983). Progesterone-Controlled Growth Hormone Overproduction and Naturally Occurring Canine Diabetes and Acromegaly. Acta Endocrinol..

[44] Bhatti S. (2006). Pituitary and Mammary Growth Hormone in Dogs. Ph.D. Thesis.

[45] Johnson C.A. (2008). Glucose Homeostasis during Canine Pregnancy: Insulin Resistance, Ketosis, and Hypoglycemia. Theriogenology.

[46] McConachie I., McConachie I. (2006). The Critically Ill Diabetic. Handbook of ICU Therapy.

[47] Panciera D.L., Di Bartola S.P. (2006). Fluid Therapy in Endocrine and Metabolic Disorders. Fluid, Electrolyte and Acid-Base Disorders in Small Animal Practice.

[48] Chiasson J.L., Aris-Jilwan N., Bélanger R., Bertrand S., Beauregard H., Ekoé J.-M., Fournier H., Havrankova J. (2003). Diagnosis and Treatment of Diabetic Ketoacidosis and the Hyperglycemic Hyperosmolar State. CMAJ.

[49] Johnson C.A., Grace J.A., Probst M.R. (1987). The Effect of Maternal Illness on Perinatal Health. Vet. Clin. N. Am. Small Anim. Pract..

[50] Fontbonne A., Siliart B., Dadinand F. (1992). Hormonal Findings in Dogs and Bitches Showing Reproductive Disorders. J. Reprod. Fertil. Suppl..

[51] Johnson C., Olivier N.B., Nachreiner R., Mullaney T. (1999). Effect of 131I-Induced Hypothyroidism on Indices of Reproductive Function in Adult Male Dogs. J. Vet. Intern. Med..

[52] Johnson C.A. (1994). Reproductive Manifestations of Thyroid Disease. Vet. Clin. N. Am. Small Anim. Pract..

[53] Johnson C.A. (2002). Thyroid Issues in Reproduction. Clin. Tech. Small Anim. Pract..

[54] Nesbitt T., Izzo G.J., Peterson L., Wilkins R.J. (1980). Canine Hypothyroidism: A Retrospective Study of 108 Cases. J. Am. Vet. Med. Assoc..

[55] Panciera D.L., Purswell B.J., Kolster K.A., Werre S.R., Trout S.W. (2012). Reproductive Effects of Prolonged Experimentally Induced Hypothyroidism in Bitches. J. Vet. Intern. Med..

[56] Panciera D.L., Purswell B.J., Kolster K.A. (2007). Effect of Short-Term Hypothyroidism on Reproduction in the Bitch. Theriogenology.

[57] Poppe K., Glinoer D. (2003). Thyroid Autoimmunity and Hypothyroidism before and during Pregnancy. Hum. Reprod. Update.

[58] Meyers-Wallen V.N. (1991). Clinical Approach to Infertile Male Dogs with Sperm in the Ejaculate. Vet. Clin. N. Am. Small Anim. Pract..

[59] Beale K.M., Bloomberg M.S., van Gilder J., Wolfson B.B., Keisling K. (1992). Correlation of Racing and Reproductive Performance in Greyhounds with Response to Thyroid Function Testing. J. Am. Anim. Hosp. Assoc..

[60] Segalini V., Hericher T., Grellet A., Rosenberg D., Garnier F., Fontbonne A. (2009). Thyroid Function and Infertility in the Dog: A Survey in Five Breeds. Reprod. Domest. Anim..

[61] Young E.J. (1983). Human Brucellosis. Rev. Infect. Dis..

[62] Wanke M.M. (2004). Canine Brucellosis. Anim. Reprod. Sci..

[63] Hollett R.B. (2006). Canine Brucellosis: Outbreaks and Compliance. Theriogenology.

[64] Brower A., Okwumabua O., Massengill C., Muenks Q., Vanderloo P., Duster M., Homb K., Kurth K. (2007). Investigation of the Spread of Brucella canis via the U.S. Interstate Dog Trade. Int. J. Infect. Dis..

[65] Holst B.S., Löfqvist K., Ernholm L., Eld K., Cedersmyg M., Hallgren G. (2012). The First Case of Brucella canis in Sweden: Background, Case Report and Recommendations from a Northern European Perspective. Acta Vet. Scand..

[66] Lucero N.E., Ayala S.M., Escobar G.I., Jacob N.R. (2008). Brucella Isolated in Humans and Animals in Latin America from 1968 to 2006. Epidemiol. Infect..

[67] Santos R.L., Souza T.D., Mol J.P.S., Eckstein C., Paíxão T.A. (2021). Canine Brucellosis: An Update. Front. Vet. Sci..

[68] Makloski C.L. (2011). Canine Brucellosis Management. Vet. Clin. N. Am. Small Anim. Pract..

[69] Souza T.D., Carvalho T.F., Mol J.P.S., Lopes J.V.M., Silva M.F., Paixão T.A., Santos R.L. (2018). Tissue Distribution and Cell Tropism of Brucella canis in Naturally Infected Canine Foetuses and Neonates. Sci. Rep..

[70] Gyuranecz M., Szeredi L., Rónai Z., Dénes B., Dencso L., Dán Á., Pálmai N., Hauser Z., Lami E., Makrai L. (2011). Detection of Brucella canis-Induced Reproductive Diseases in a Kennel. J. Vet. Diagn. Investig..

[71] Carmichael L.E., Kenney R.M. (1968). Canine Abortion Caused by Brucella canis. J. Am. Vet. Med. Assoc..

[72] Carvalho Neta A.V., Mol J.P., Xavier M.N., Paixão T.A., Lage A.P., Santos R.L. (2010). Pathogenesis of Bovine Brucellosis. Vet. J..

[73] Poester F.P., Samartino L.E., Santos R.L. (2013). Pathogenesis and Pathobiology of Brucellosis in Livestock. Rev. Sci. Tech..

[74] Moore J.A., Gupta B.N. (1970). Epizootiology, Diagnosis, and Control of Brucella canis. J. Am. Vet. Med. Assoc..

[75] Olsen S.C., Palmer M.V. (2014). Advancement of Knowledge of Brucella Over the Past 50 Years. Vet. Pathol..

[76] George L.W., Duncan J.R., Carmichael L.E. (1979). Semen Examination in Dogs with Canine Brucellosis. Am. J. Vet. Res..

[77] Anderson G.I., Binnington A.G. (1983). Discospondylitis and Orchitis Associated with High Brucella Titre in a Dog. Can. Vet. J..

[78] Kerwin S.C., Lewis D.D., Hribernik T.N., Partington B., Hosgood G., Eilts B.E. (1992). Diskospondylitis Associated with Brucella canis Infection in Dogs: 14 Cases (1980–1991). J. Am. Vet. Med. Assoc..

[79] Ledbetter E.C., Landry M.P., Stokol T., Kern T.J., Messick J.B. (2009). Brucella canis Endophthalmitis in 3 Dogs: Clinical Features, Diagnosis, and Treatment. Vet. Ophthalmol..

[80] Bricker B.J. (2002). PCR as a Diagnostic Tool for Brucellosis. Vet. Microbiol..

[81] George L.W., Carmichael L.E. (1974). A Plate Agglutination Test for the Rapid Diagnosis of Canine Brucellosis. Am. J. Vet. Res..

[82] Badakhsh F., Carmichael L., Douglass J. (1982). Improved Rapid Slide Agglutination Test for Presumptive Diagnosis of Canine Brucellosis. J. Clin. Microbiol..

[83] Lucero N.E., Escobar G.I., Ayala S.M., Lopez G. (2002). Sensitivity and Specificity of an Indirect Enzyme-Linked Immunoassay for the Diagnosis of Brucella canis Infection in Dogs. J. Med. Microbiol..

[84] de Oliveira M.Z., Vale V., Keid L., Freire S.M., Meyer R., Portela R.W., Barrouin-Melo S.M. (2011). Validation of an ELISA Method for the Serological Diagnosis of Canine Brucellosis Due to Brucella canis. Res. Vet. Sci..

[85] Wanke M., Cairó F., Rossano M., Laiño M., Baldi P.C., Monachesi N.E., Comercio E., Vivot M. (2012). Preliminary Study of an Immunochromatography Test for Serological Diagnosis of Canine Brucellosis. Reprod. Domest. Anim..

[86] Barkha S., Kumar S.D., Kumar S.D. (2011). Immunochemical Characterization of Antigens of Brucella canis and Their Use in Seroprevalence Study of Canine Brucellosis. Asian Pac. J. Trop. Med..

[87] Cosford K.L. (2018). Brucella canis: An Update on Research and Clinical Management. Can. Vet. J..

[88] Mateu-de-Antonio E.M., Martín M. (1995). In Vitro Efficacy of Several Antimicrobial Combinations Against Brucella canis and Brucella melitensis Strains Isolated from Dogs. Vet. Microbiol..

[89] Zoha S.J., Walsh R. (1982). Effect of a Two-Stage Antibiotic Treatment Regimen on Dogs Naturally Infected with Brucella canis. J. Am. Vet. Med. Assoc..

[90] Bulgin M.S., Ward A.C., Sriranganathan N., Saras P. (1984). Abortion in the Dog Due to Campylobacter Species. Am. J. Vet. Res..

[91] Odendaal M.W., de Cramer K.G., van der Walt M.L., Botha A.D., Pieterson P.M. (1994). First Isolation of Campylobacter jejuni from the Vaginal Discharge of Three Bitches after Abortion in South Africa. Onderstepoort J. Vet. Res..

[92] Sahin O., Burrough E.R., Pavlovic N., Frana T.S., Madson D.M., Zhang Q. (2014). Campylobacter jejuni as a Cause of Canine Abortions in the United States. J. Vet. Diagn. Investig..

[93] Davies R.H., Lawes J.R., Wales A.D. (2019). Raw Diets for Dogs and Cats: A Review, with Particular Reference to Microbiological Hazards. J. Small Anim. Pract..

[94] Graham E.M., Taylor D.J. (2012). Bacterial Reproductive Pathogens of Cats and Dogs. Vet. Clin. N. Am. Small Anim. Pract..

[95] Chaban B., Musil K.M., Himsworth C.G., Hill J.E. (2009). Development of CPN-Based Real-Time Quantitative PCR Assays for the Detection of 14 Campylobacter Species and Application to Screening of Canine Fecal Samples. Appl. Environ. Microbiol..

[96] Dróżdż M., Małaszczuk M., Paluch E., Pawlak A. (2021). Zoonotic Potential and Prevalence of Salmonella Serovars Isolated from Pets. Infect. Ecol. Epidemiol..

[97] Redwood D.W., Bell D.A. (1983). Salmonella Panama: Isolation from Aborted and Newborn Canine Fetuses. Vet. Rec..

[98] Ahmed A., Ellis C., Kelleman A. (2023). Pregnancy and Spontaneous Partial Abortion Associated with Salmonella in a Dog. Clin. Theriogenol..

[99] Allen-Durrance A., Mazzaccari K.M., Woliver C.L. (2022). Bacteremia and Late-Term Abortion Secondary to Salmonellosis in a Dog. J. Am. Anim. Hosp. Assoc..

[100] Caldow G.L., Graham M.M. (1998). Abortion in Foxhounds and a Ewe Flock Associated with Salmonella montevideo Infection. Vet. Rec..

[101] Cobb M.A., Stavisky J., Barrow P.A., Methner U. (2013). Salmonella Infections in Dogs and Cats. Salmonella in Domestic Animals.

[102] Leps A.S., Klein B., Schneider M., Meyer C., Šoba A., Simon C., Dyachenko V., Siesenop U., Verspohl J., Goericke-Pesch S. (2024). The Canine Vaginal Flora: A Large-Cohort Retrospective Study. Vet. Sci..

[103] Bjurstrom L. (1993). Aerobic Bacteria Occurring in the Vagina of Bitches with Reproductive Disorders. Acta Vet. Scand..

[104] Hagman R. (2022). Pyometra in Small Animals 2.0. Vet. Clin. N. Am. Small Anim. Pract..

[105] Linde C. (1983). Partial Abortion Associated with Genital Escherichia coli Infection in a Bitch. Vet. Rec..

[106] Penzhorn B.L. (1985). Necrosis and Abscessation of Placental Sites in a Pekingese Bitch. J. S. Afr. Vet. Assoc..

[107] Allen W.E., Dagnall G.J.R. (1982). Some Observations on the Aerobic Bacterial Flora of the Genital Tract of the Dog and Bitch. J. Small Anim. Pract..

[108] Bjurstrom L., Linde-Forsberg C. (1992). Long-Term Study of Aerobic Bacteria of the Genital Tract in Breeding Bitches. Am. J. Vet. Res..

[109] Golinska E., Sowińska N., Tomusiak-Plebanek A., Szydło M., Witka N., Lenarczyk J., Strus M. (2021). The Vaginal Microflora Changes in Various Stages of the Estrous Cycle of Healthy Female Dogs and the Ones with Genital Tract Infections. BMC Vet. Res..

[110] Groppetti D., Pecile A., Barbero C., Martino P.A. (2012). Vaginal Bacterial Flora and Cytology in Proestrous Bitches: Role on Fertility. Theriogenology.

[111] Mantovani A., Restani R., Sciarra D., Simonella P. (1961). Streptococcus L Infection in the Dog. J. Small Anim. Pract..

[112] Miller C.W., Prescott J.F., Mathews K.A., Betschel S.D., Yager J.A., Guru V., DeWinter L., Low D.E. (1996). Streptococcal Toxic Shock Syndrome in Dogs. J. Am. Vet. Med. Assoc..

[113] DeWinter L.M., Prescott J.F. (1999). Relatedness of Streptococcus canis from Canine Streptococcal Toxic Shock Syndrome and Necrotizing Fasciitis. Can. J. Vet. Res..

[114] Lamm C.G., Ferguson A.C., Lehenbauer T.W., Love B.C. (2010). Streptococcal Infection in Dogs: A Retrospective Study of 393 Cases. Vet. Pathol..

[115] Low J.C., Donachie W. (1997). A Review of Listeria monocytogenes and Listeriosis. Vet. J..

[116] Fenwick B., Osburn B.I. (1986). Listeriosis in Animals. Vet. Clin. N. Am. Food Anim. Pract..

[117] Guillet C., Join-Lambert O., Le Monnier A., Leclercq A., Mechaï F., Mamzer-Bruneel M.-F., Bielecka M.K., Scortti M., Disson O., Berche P. (2012). Human Listeriosis Caused by Listeria ivanovii. Emerg. Infect. Dis..

[118] Sturgess C.P. (1989). Listerial Abortion in the Bitch, Editorial Comment. Vet. Rec..

[119] Pretzer S.D. (2008). Leptospirosis and Reproductive Disease. Veterinary Reproduction and Obstetrics.

[120] Ellis W.A. (1986). Leptospirosis. J. Small Anim. Pract..

[121] Goldstein R.E. (2010). Canine Leptospirosis. Vet. Clin. N. Am. Small Anim. Pract..

[122] Langston C.E., Heuter K.J. (2003). Leptospirosis: A Re-emerging Zoonotic Disease. Vet. Clin. N. Am. Small Anim. Pract..

[123] Schuller S., Francey T., Hartmann K., Hugonnard M., Kohn B., Nally J.E., Sykes J. (2015). European Consensus Statement on Leptospirosis in Dogs and Cats. J. Small Anim. Pract..

[124] Doig P.A., Ruhunke H.L., Bosu W.T.K. (1981). The Genital Mycoplasma and Ureaplasma of Healthy and Diseased Dogs. Can. J. Comp. Med..

[125] Feldman E.C., Nelson R.W., Kersey R. (2004). Periparturient Diseases. Canine and Feline Endocrinology and Reproduction.

[126] Lein D.H. (1986). Canine Mycoplasma, Ureaplasma, and Bacterial Infertility. Curr. Vet. Ther. Small Anim. Pract..

[127] Holzmann A., Laber G., Walzl H. (1979). Experimentally Induced Mycoplasmal Infection in the Genital Tract of the Female Dog. Theriogenology.

[128] L’Abee-Lund T.M., Heiene R., Friis N.F., Ahrens P., Sørum H. (2003). Mycoplasma canis and Urogenital Disease in Dogs in Norway. Vet. Rec..

[129] Furthner E., Maenhoudt C., Boucher C., Fontbonne A. (2018). Embryonic Resorptions and Neonatal Mortality in a Canine Kennel with Identification of Uterine Mycoplasma. Reprod. Domest. Anim..

[130] Tamiozzo P.J. (2022). Mycoplasma maculosum and Mycoplasma spumans Associated with Fertility Disorders in Dogs from a Bernese Mountain Dog Kennel. Rev. Argent. Microbiol..

[131] Post K. (1995). Embryo and Fetal Loss in the Canine: A Review. Theriogenology Handbook.

[132] Spergser J., Schäfer-Somi S., Mantziaras G., Arlt S. (2018). Mycoplasma in Dogs: Significance, Diagnosis and Treatment. Proceedings of the 21th EVSSAR Congress.

[133] Chalker V.J. (2005). Canine Mycoplasmas. Res. Vet. Sci..

[134] Johnston S.D., Kustritz M.V.R., Olson P.N.S., Kersey R. (2001). Disorders of the Canine Testes and Epididymis. Canine and Feline Theriogenology.

[135] Chamberlain D.M., Doctor F.L., Cole C.R. (1953). Toxoplamosis. II. Intrauterine Infection in Dogs, Premature Birth and Presence of Organisms in Milk. Proc. Soc. Exp. Biol. Med..

[136] Dubey J.P., Lindsay D.S. (1989). Transplacental *Neospora caninum* Infection in Dogs. Am. J. Vet. Res..

[137] Dubey J.P., Koestner A., Piper R.C. (1990). Repeated Transplacental Transmission of *Neospora caninum* in Dogs. J. Am. Vet. Med. Assoc..

[138] Barber J.S., Trees A.J. (1998). Naturally Occurring Vertical Transmission of *Neospora caninum* in Dogs. Int. J. Parasitol..

[139] Barber J.S., Gasser R.B., Ellis J., Reichel M.P., McMillan D., Trees A.J. (1998). Prevalence of Antibodies to *Neospora caninum* in Different Canid Populations. J. Parasitol..

[140] Dubey J.P. (1999). *Neospora caninum* and Neosporosis in Animals. Korean J. Parasitol..

[141] Dubey J.P., Lindsay D.S. (1996). A Review of *Neospora caninum* and Neosporosis. Vet. Parasitol..

[142] Dubey J.P., Rosypal A.C., Pierce V., Scheinberg S.N., Lindsay D.S. (2005). Placentitis Associated with Leishmaniasis in a Dog. J. Am. Vet. Med. Assoc..

[143] Miró G., Cardoso L., Pennisi M.G., Oliva G., Baneth G. (2013). Canine Leishmaniosis—New Concepts and Insights on an Expanding Zoonosis: Part Two. Trends Parasitol..

[144] Maia C., Campino L. (2011). Canine Leishmaniasis: An Overview of the Current Status and Strategies for Control. Vet. Parasitol..

[145] Solano-Gallego L., Miró G., Koutinas A., Cardoso L., Pennisi M.G., Ferrer L., Bourdeau P., Oliva G., Baneth G. (2011). LeishVet Guidelines for the Practical Management of Canine Leishmaniosis. Parasitol..

[146] Binn L.N., Lazar E.C., Eddy G.A. (1970). Recovery and Characterization of a Minute Virus of Canines. Infect. Immun..

[147] Manteufel J., Truyen U. (2008). Parvovirus Infections in Animals. Vet. Microbiol..

[148] Decaro N., Buonavoglia C. (2012). Canine Parvovirus Type 1 and Its Role in Canine Health. Vet. Microbiol..

[149] Harrison G. (1992). Parvoviral Myocarditis in Puppies. Vet. Clin. N. Am. Small Anim. Pract..

[150] Carmichael L.E., Schlafer D.H. (1994). Clinical and Pathological Features of CPV-1 Infection. Vet. Pathol..

[151] Eminaga S., Martinez N., Fraga M., Tebar M., Ferrer D., Addie D., Cerón J.J., Martínez-Subiela S. (2011). CPV-1 Infection in Young Puppies. J. Small Anim. Pract..

[152] Manteufel J., Truyen U. (2008). Animal Bocaviruses: A Brief Review. Intervirology.

[153] Carmichael L.E., Schlafer D.H., Hashimoto A. (1991). Canine Parvovirus Type-1 and Reproductive Failure. J. Vet. Med..

[154] Decaro N., Carmichael L.E., Buonavoglia C. (2012). Viral Reproductive Pathogens of Dogs and Cats. Vet. Clin. N. Am. Small Anim. Pract..

[155] Truyen U., Evermann J.F. (1996). CPV-1 and Abortion in Dogs: Case Reports and Literature Review. Vet. Rec..

[156] Mochizuki M., Hashimoto M., Matsui O. (2002). Immunofluorescence and PCR as Diagnostic Tools for CPV-1. Jpn. J. Vet. Sci..

[157] Decaro N., Martella V., Elia G. (2008). Diagnostic Techniques in CPV-1 Detection. J. Clin. Microbiol..

[158] Squires E.L. (2024). Vaccination Strategies in Breeding Animals. Veterinary Reproduction and Obstetrics.

[159] Carmichael L.E., Squire A., Krook L. (1965). Clinical and Pathological Features of a Fatal Viral Disease of Newborn Pups. Am. J. Vet. Res..

[160] Decaro N., Martella V., Buonavoglia C. (2008). Canine Adenoviruses and Herpesvirus. Vet. Clin. N. Am. Small Anim. Pract..

[161] Carmichael L.E. (1970). Herpesvirus Canis: Aspects of Pathogenesis and Immune Response. J. Am. Vet. Med. Assoc..

[162] Poste G., King N. (1971). Isolation of a Herpesvirus from the Canine Genital Tract: Association with Infertility, Abortion, and Stillbirths. Vet. Rec..

[163] Hashimoto A., Hirai K., Okada K. (1979). Pathology of the Placenta and Newborn Pups with Suspected Intrauterine Infection of Canine Herpesvirus. Am. J. Vet. Res..

[164] Nauwynck H., Rijsselaere T., Luvoni G.C., Bogaerts P. (2010). Canine Herpes Virus 1-Infections in Dogs: Truth and Lies. Proceedings of the 7th EVSSAR Congress.

[165] Decaro N., Amorisco F., Desario C. (2010). Development and Validation of a Real-Time PCR Assay for Specific and Sensitive Detection of Canid Herpesvirus 1. J. Virol. Methods.

[166] Mir F., Fontaine E., Albaric O., Greer M., Vannier F., Schlafer D.H., Fontbonne A. (2013). Findings in Uterine Biopsies Obtained by Laparotomy from Bitches with Unexplained Infertility or Pregnancy Loss: An Observational Study. Theriogenology.

[167] Poulet H., Guigal P.M., Soulier M., Leroy V., Fayet G., Minke J., Chappuis G. (2001). Protection of Puppies against Canine Herpesvirus by Vaccination of the Dams. Vet. Rec..

[168] Krakowka S., Hoover E.A., Koestner A., Ketring K. (1977). Experimental and Naturally Occurring Transplacental Transmission of Canine Distemper Virus. Am. J. Vet. Res..

[169] Verstegen J. (2008). The Effects of Canine Distemper Virus on Maternal and Fetal Health. J. Vet. Intern. Med..

[170] Fontbonne A. (2023). Reproductive Impacts of Canine Distemper Virus. Vet. Clin. N. Am. Small Anim. Pract..

[171] Martella V., Elia G., Buonavoglia C. (2008). Canine Distemper Virus. Vet. Clin. N. Am. Small Anim. Pract..

[172] Squires R.A., Crawford C., Marcondes M., Whitley N. (2024). 2024 Guidelines for the Vaccination of Dogs and Cats–Compiled by the Vaccination Guidelines Group (VGG) of the World Small Animal Veterinary Association (WSAVA). J. Small Anim. Pract..

[173] Verstegen J. (2008). Canine Adenovirus and Reproductive Complications. J. Vet. Intern. Med..

[174] Fontbonne A. (2023). Impact of Canine Adenovirus on Reproductive Health. Vet. Clin. N. Am. Small Anim. Pract..

[175] Almes J., Little P. (2010). Fluorescent Antibody and PCR Methods for the Diagnosis of Canine Adenovirus Infections. J. Vet. Diagn. Investig..

[176] Schlafer D. (2012). Diseases of the Canine Uterus. Reprod. Domest. Anim..

[177] Hagman R. (2012). Clinical and Molecular Characteristics of Pyometra in Female Dogs. Reprod. Domest. Anim..

[178] Hagman R. (2014). Diagnostic and Prognostic Markers for Uterine Diseases in Dogs. Reprod. Domest. Anim..

[179] Hagman R. (2018). Pyometra in Small Animals. Vet. Clin. N. Am. Small Anim. Pract..

[180] Bigliardi E., Parmigiani E., Cavirani S., Luppi A., Bonati L., Corradi A. (2004). Ultrasonography and Cystic Hyperplasia-Pyometra Complex in the Bitch. Reprod. Domest. Anim..

[181] Woźna-Wysocka M., Rybska M., Błaszak B., Nowicki M., Dzięgiel P. (2021). Morphological Changes in Bitches Endometrium Affected by Cystic Endometrial Hyperplasia-Pyometra Complex—The Value of Histopathological Examination. BMC Vet. Res..

[182] Quartuccio M., Liotta L., Cristarella S., Lanteri G., Ieni A., D’Arrigo T., De Majo M. (2020). Contrast-Enhanced Ultrasound in Cystic Endometrial Hyperplasia–Pyometra Complex in the Bitch: A Preliminary Study. Animals.

[183] Schlafer D.H., Gifford A.T. (2008). Cystic Endometrial Hyperplasia, Pseudo-Placentational Endometrial Hyperplasia, and Other Cystic Conditions of the Canine and Feline Uterus. Theriogenology.

[184] Gifford A.T., Scarlett J.M., Schlafer D.H. (2014). Histopathologic Findings in Uterine Biopsy Samples from Subfertile Bitches: 399 Cases (1990–2005). J. Am. Vet. Med. Assoc..

[185] Younis M., Mohammed F.F., Ragab R.S., Gohar H.M. (2014). Ultrasonography and Pathological Evaluation of Cystic Endometrial Hyperplasia Pyometra Complex in Bitches and Queens with Related Ovarian Alterations. Glob. Vet..

[186] Christensen B., Schlafer D., Agnew D., Wang C., Kozlowski C., Asa C. (2012). Diagnostic Value of Transcervical Endometrial Biopsies in Domestic Dogs Compared with Full-Thickness Uterine Sections. Reprod. Domest. Anim..

[187] Günzel-Apel A.R., Wilke M., Aupperle H., Schoon H.A. (2001). Development of a Technique for Transcervical Collection of Uterine Tissue in Bitches. J. Reprod. Fertil. Suppl..

[188] Groppetti D., Pecile A., Arrighi S., Di Giancamillo A., Cremonesi F. (2010). Endometrial Cytology and Computerized Morphometric Analysis of Epithelial Nuclei: A Useful Tool for Reproductive Diagnosis in the Bitch. Theriogenology.

[189] Fontaine E., Levy X., Grellet A., Luc A., Bernex F., Boulouis H.J., Fontbonne A. (2009). Diagnosis of Endometritis in the Bitch: A New Approach. Reprod. Domest. Anim..

[190] Papich M.G., Kirk R.W. (1989). Effects of Drugs on Pregnancy. Current Veterinary Therapy X.

[191] Wiebe V.J., Howard J.P. (2009). Pharmacologic Advances in Canine and Feline Reproduction. Top. Companion Anim. Med..

[192] Blythe S.A., England G.C.W. (1993). Effect of Age upon Reproductive Efficiency in the Bitch. J. Reprod. Fertil. Suppl..

[193] Chastant-Maillard S., Guillemot C., Feugier A., Mariani C., Grellet A., Mila H. (2017). Reproductive Performance and Pre-Weaning Mortality: Preliminary Analysis of 27,221 Purebred Female Dogs and 204,537 Puppies in France. Reprod. Domest. Anim..

[194] Gavrilovic B.B., Andersson K., Linde-Forsberg C. (2008). Reproductive patterns in the domestic dog—A retrospective study of the Drever breed. Theriogenology.

[195] Baalbergen T., de Gier J., van Steenbeek F.G. (2021). Ovulation timing in the bitch: Conception rate and influencing factors in 1401 estrus cycles. PhD Thesis.

[196] Uchańska O., Ochota M., Eberhardt M., Niżański W. (2022). Dead or Alive? A Review of Perinatal Factors That Determine Canine Neonatal Viability. Animals.

[197] Berepubo N.A., Long S.E. (1983). A study of the relationship between chromosome anomalies and reproductive wastage in domestic animals. Theriogenology.

[198] Romagnoli S., Schäfer-Somi S., Podhajsky E., Günzel-Apel A.-R., Hagman R. (2015). When Pregnancy Is Jeopardized—Causes and Therapeutical Options. Proceedings of the 18th EVSSAR Congress.

[199] Tal S., Bar-Gal G.K., Arlt S.P. (2021). Evaluation of Short-Term Safety of Ultrasound-Guided Foetal Fluid Sampling in the Dog (*Canis lupus familiaris*). Vet. Rec..

[200] Tal S., Sutton G., Arlt S., Bar-Gal G.K. (2022). Analysis of Biochemical Parameters in Canine Fetal Fluids during the Second Half of Pregnancy. Theriogenology.

